# 

**Published:** 1878-08

**Authors:** 


### Table of Contents.

### ORIGINAL COMMUNICATIONS.

STRICTURES OF THE CERVICAL CANAL, and of the Internal and External Os—Translation from the Swedish. *By A. Sibley Campbell, M.D., Augusta, Ga.* ..... 257

PROGRESS OF GYNÆCOLOGY. *By J. G. Westmoreland, M.D., Atlanta, Ga.* ..... 302

### EDITORIAL.

Yellow Fever ..... 308

Metric System ..... 309

### BIBLIOGRAPHICAL.

The Science and Art of Surgery ..... 310

A Manual of Operative Surgery ..... 311

Nervous Diseases ..... 311

Fowne's Manual of Chemistry ..... 311

Hand-Book of Ophthalmology ..... 312

Pamphlets Received ..... 312

College Announcements Received ..... 313

### EXCERPTA.

Indian Obstetrics ..... 313

Substitute for Calomel ..... 315

Pneumatic Forceps ..... 316

Atropia in Epilepsy ..... 316

Sunstroke ..... 316

Treatment of Enlarged Prostate ..... 317

Treatment of Hydrocele by Incision Performed Antiseptically ..... 318

Lactopeptine ..... 318

Thymol, the New Antiseptic ..... 319

To Subscribers in Arrears ..... 320

